# Exosomes Secreted by Wharton’s Jelly-Derived Mesenchymal Stem Cells Promote the Ability of Cell Proliferation and Migration for Keratinocyte

**DOI:** 10.3390/ijms25094758

**Published:** 2024-04-26

**Authors:** Hong-Ren Yu, Hsin-Chun Huang, I-Lun Chen, Sung-Chou Li

**Affiliations:** 1 Department of Pediatrics, Kaohsiung Chang Gung Memorial Hospital, Chang Gung University College of Medicine, Kaohsiung 833401, Taiwan; yuu2004taiwan@yahoo.com.tw (H.-R.Y.); hhuang@cgmh.org.tw (H.-C.H.); memeo103@hotmail.com (I.-L.C.); 2 Department of Medical Education and Research, Kaohsiung Veterans General Hospital, Kaohsiung 813414, Taiwan; 3 Department of Dental Technology, Shu-Zen Junior College of Medicine and Management, Kaohsiung 821004, Taiwan

**Keywords:** Wharton’s jelly mesenchymal stem cell, adipose tissue mesenchymal stem cell, exosome, iTRAQ, fibrinogen beta chain

## Abstract

Mesenchymal stem cells (MSCs) isolated from Wharton’s jelly (WJ-MSCs) and adipose tissue (AD-MSCs) are alternative sources for bone marrow-derived MSCs. Owing to their multiple functions in angiogenesis, immune modulation, proliferation, migration, and nerve regeneration, MSC-derived exosomes can be applied in “cell-free cell therapy”. Here, we investigated the functional protein components between the exosomes from WJ-MSCs and AD-MSCs to explain their distinct functions. Proteins of WJ-MSC and AD-MSC exosomes were collected and compared based on iTRAQ gel-free proteomics data. Results: In total, 1695 proteins were detected in exosomes. Of these, 315 were more abundant (>1.25-fold) in AD-MSC exosomes and 362 kept higher levels in WJ-MSC exosomes, including fibrinogen proteins. Pathway enrichment analysis suggested that WJ-MSC exosomes had higher potential for wound healing than AD-MSC exosomes. Therefore, we treated keratinocyte cells with exosomes and the recombinant protein of fibrinogen beta chain (FGB). It turned out that WJ-MSC exosomes better promoted keratinocyte growth and migration than AD-MSC exosomes. In addition, FGB treatment had similar results to WJ-MSC exosomes. The fact that WJ-MSC exosomes promoted keratinocyte growth and migration better than AD-MSC exosomes can be explained by their higher FGB abundance. Exploring the various components of AD-MSC and WJ-MSC exosomes can aid in their different clinical applications.

## 1. Introduction

Mesenchymal stem cells (MSCs) are undifferentiated cells with a high proliferation capacity and mesodermal differentiation potential [1]. They are an important source of stem cells for damaged tissue regeneration in clinical applications. Although bone marrow (BM) has conventionally been used as the major source of pluripotent MSCs, BM collection requires a highly invasive procedure. Furthermore, with increasing age, the number, differentiation potential, and lifespan of MSCs from BM decrease [2]. Therefore, the umbilical cord and adipose tissue (AD) are used as alternative sources of MSCs [2]. Umbilical cord-derived stem cells originating in Wharton’s jelly (WJ) are called Wharton’s jelly mesenchymal stem cells (WJ-MSCs). They form a class of stem cells with a high differentiation potential, an immuno-privileged status, and easy access for collection, raising no legal or ethical issues. WJ-MSCs exhibit several features of embryonic stem cells, such as a short doubling time and potent expansion capacity, with only a few differences [3].

Although all mesenchymal stem cells (MSCs) exhibit similar phenotypes to one another [4], based on experimental results, it is evident that WJ-MSCs exhibit higher proliferation potential compared to adipose tissue-derived mesenchymal stem cells (AD-MSCs) [4,5]. Both cell types can differentiate into adipocytes, osteoblasts, and chondrocytes, but AD-MSCs demonstrate a higher adipogenicity potential than WJ-MSCs. WJ-MSCs secrete higher levels of RANTES, IP-10, MCP-1, IL-6, and IL-8 compared to AD-MSCs, while AD-MSCs secrete more IL-7; IL-12; collagen types I, II, III, IV, and V; and VEGF than WJ-MSCs [5]. These results reaffirm the pro-inflammatory characteristics of WJ-MSCs. This underscores that WJ-MSCs possess higher pro-inflammatory and proliferative properties, while AD-MSCs secrete higher concentrations of angiogenic proteins and extracellular matrix components. The distinct properties of mesenchymal cells from different sources in promoting regeneration, angiogenesis, or anti-inflammatory activity suggest their potential differentiation ability in various clinical applications. Owing to their immune modulatory capacity, WJ-MSCs have been applied in the management of autoimmune and graft-versus-host diseases [3,6]. Meanwhile, adipose tissue-derived mesenchymal stem cells (AD-MSCs) exhibit self-renewal potential and multipotential differentiation ability. This multipotentiality of AD-MSCs promotes their application in regenerative medicine [7]. However, several limitations to stem cell therapy remain, including low survival rate, immunological rejection, and carcinogenic risk [8,9].

Exosomes are small vesicles (50–150 nm) released into the extracellular space after the fusion of multivesicular bodies with the cell membrane. Exosomes are composed of a lipid bilayer coated with transmembrane proteins, which encloses cytosolic proteins, mRNAs, microRNAs, and other non-coding RNAs [10,11,12]. After attaching to the target cell, extracellular vesicles induce downstream signaling through receptor–ligand interactions, internalization via endocytosis/phagocytosis, or fusion with the target cell membrane to deliver their content into the target cell’s cytosol, thereby affecting the recipient cell [13]. Exosomes can be secreted by many types of cells [14,15,16,17]. Intercellular communication regulating the functions of exosomes, such as angiogenesis, immune modulation, proliferation, migration, and nerve regeneration, has been documented [18]. Owing to their multiple functions, exosomes hold immense therapeutic potential, including cutaneous healing and regeneration, neuroprotection and neurodegeneration (e.g., in Alzheimer’s disease, Huntington’s disease, and amyotrophic lateral sclerosis), and ischemia-reperfusion rescue (e.g., skin flap, heart, and kidney) [18]. In addition, owing to their excellent material transportation properties, intrinsic long-term circulatory capability, and biocompatibility, exosomes have been utilized for targeted drug delivery and as gene carriers in regenerative medicine. Thanks to their outstanding characteristics, exosomes can be applied in “cell-free cell therapy”.

Given the differences between WJ-MSCs and AD-MSCs, the exosomes derived from these sources are expected to be dissimilar. Differences across MSCs derived from various tissues and their exosomes have already been explored in previous studies [6,19,20]. However, these studies either provided differential protein profiles or performed limited functional comparisons. To this end, the present study aimed to provide a deeper understanding on the differences between WJ-MSC- and AD-MSC-derived exosomes, together with functional validation.

## 2. Results

### 2.1. Discrimination of Exosomes Derived from AD-MSCs and WJ-MSCs

**Figure 1:**
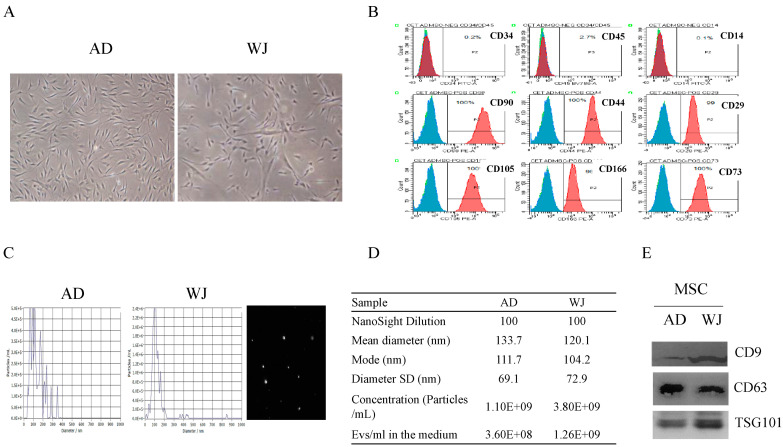
Morphologies, surface markers, and characteristics of AD-MSCs and WJ-MSCs and their exosomes. (**A**) Morphology of AD-MSCs and WJ-MSCs. (**B**) The common surface markers of AD-MSCs and WJ-MSCs, plotted based on by AD-MSCs. (**C**,**D**) Exosomes secreted by MSCs were confirmed with nanoparticle tracking analysis and (**E**) Western blotting. Abbreviations: AD, adipose tissue-derived mesenchymal stem cells; WJ, Wharton’s jelly-derived mesenchymal stem cells; MSC, mesenchymal stem cells.

We cultured AD-MSCs and WJ-MSCs and collected exosomes derived from both types of cells. Figure 1A illustrates the morphology of AD-MSCs and WJ-MSCs under the microscope. Both cells were spindle-shaped. In addition, both AD-MSCs and WJ-MSCs presented positive signals for surface markers CD90, CD44, CD29, CD105, CD166, and CD73 but negative signals for surface markers CD34, CD14, and CD45; a representative image is shown in Figure 1B. The size of both AD-MSCs and WJ-MSCs was variable (Figure 1C). In addition, exosomes secreted by AD-MSCs and WJ-MSCs were confirmed. The quantity and quality were controlled with nanoparticle tracking analysis (NTA) (Figure 1D). Both AD-MSC and WJ-MSC exosomes exhibited specific exosomes markers (CD9, CD63, and TSG101; Figure 1E).

### 2.2. Protein Abundance Profiles of AD-MSC and WJ-MSC Exosomes

**Figure 2:**
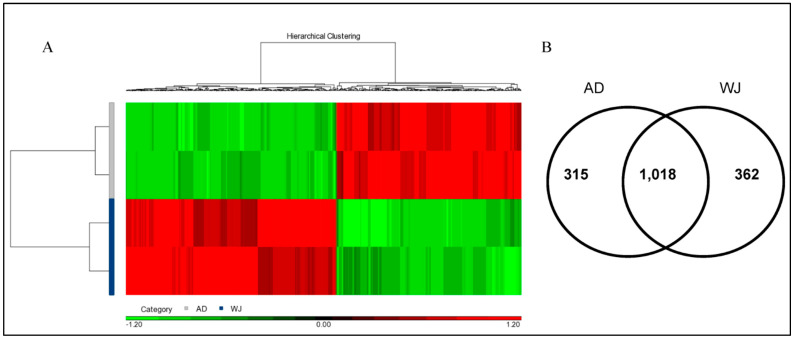
Heat map and Venn diagram of differentially abundant proteins extracted from exosomes of AD-MSCs and WJ-MSCs. (**A**) The heat map generated based on the 677 differential abundant proteins. (**B**) The Venn diagram illustrates the number of up-regulated proteins in the exosomes of AD-MSCs and WJ-MSCs.

Next, we applied iTRAQ gel-free proteomics to compare the protein abundances between AD and WJ exosomes. Based on two parameters, protein and peptide identification with the false discovery rate (FDR) of <0.01 and at least two unique peptides (UP ≥ 2), we detected 1695 proteins in AD-MSC and WJ-MSC exosomes (detailed protein abundance table in Supplementary Table S1). Using two AD-MSC and two WJ-MSC exosome protein samples, we compared the protein abundances with Partek and ANOVA. This demonstrated that 677 proteins were within >1.25-fold variation between AD-MSC and WJ-MSC exosomes (Figure 2A), and they could be well-separated based on the abundance profile. Among them, 315 and 362 proteins were more abundant in AD-MSC and WJ-MSC exosomes, respectively (Figure 2B, detailed *p*-value and variation ratio are available in Supplementary Table S2). The remaining 1018 proteins were consensually non-varying between the two types of exosomes.

### 2.3. Enrichment of AD-MSC and WJ-MSC Exosome-Specific Proteins

To investigate the possible functions of differential or consensus proteins between AD-MSC and WJ-MSC exosomes (three sets of proteins in Figure 2B), we performed KEGG pathway enrichment analyses on three sets of genes. Table 1 lists the top ten most significant pathways of the consensus proteins (the intersected 1018 proteins), including ribosome, proteasome, bacterial invasion of epithelial cells, carbon metabolism, and protein processing in endoplasmic reticulum. The top five most significant pathways for WJ-MSC exosome-specific proteins were complement and coagulation cascades, ECM–receptor interaction, focal adhesion, amoebiasis, and proteasome. Meanwhile, Parkinson’s disease, protein digestion and absorption, cardiac muscle contraction, Alzheimer’s disease, and oxidative phosphorylation pathways were the top five most significant pathways of AD-MSC exosome-specific proteins. These results imply differences in the clinical applications of AD-MSC and WJ-MSC exosomes. Overall, our iTRAQ gel-free proteomics analysis provided a reliable dataset deserving further investigation.

### 2.4. Abundance of Fibrinogen Proteins in WJ-MSC Exosomes

**Figure 3:**
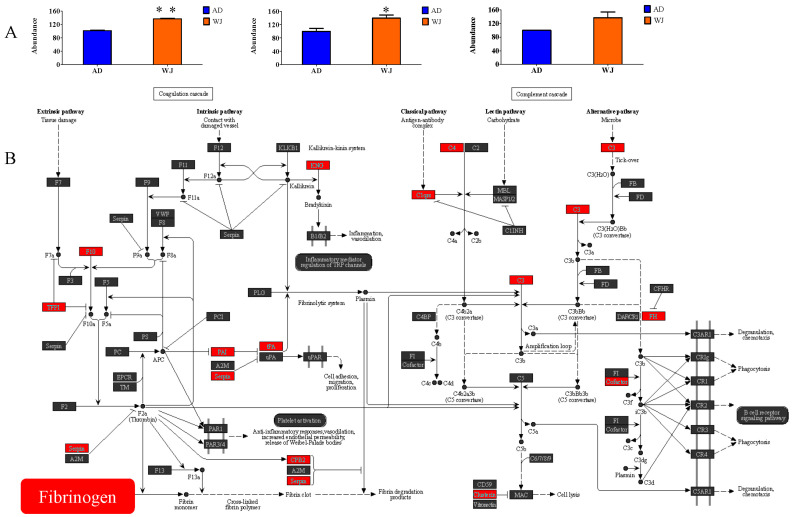
Abundance of fibrinogen-related proteins and their relevant regulatory roles in WJ-MSC exosomes. (**A**) Relative abundances of FGA (left), FGB (middle), and FGG (right) in AD-MSC and WJ-MSC exosomes (two repetitions; for each repetition, duplicates were used). The abundance values were acquired from iTRAQ assay. * and ** denoted *p* < 0.05 and *p* < 0.01, respectively. (**B**) Fibrinogen and its related proteins in the complement and coagulation cascade pathway. Red boxes represent the more abundant proteins in WJ-MSC exosomes than in AD-MSC exosomes. The stronger the red, the more abundant the molecular protein. The grey boxes denoted the proteins without abundance variation. Abbreviations: AD-MSCs, adipose tissue-derived mesenchymal stem cells; WJ-MSCs, Wharton’s jelly-derived mesenchymal stem cells; FGA, fibrinogen alpha chain; FGB, fibrinogen beta chain; FGG, fibrinogen gamma chain.

Among the 677 differentially abundant proteins, we focused on fibrinogen proteins in exosomes. Due to its high biocompatibility with fibrinogen, bioactive molecules conjugated with fibrinogen can promote tissue morphogenesis or maturation after cell adhesion, migration, proliferation, or differentiation on the substrate. Therefore, fibrinogen is widely used in regenerative medicine related to cardiac tissue regeneration and repair of bone or nerve injuries [21,22,23,24]. Moreover, WJ-MSC exosome-specific proteins were the most significantly enriched in complement and coagulation cascade pathways. Therefore, we first compared the abundances of fibrinogen alpha chain (FGA), fibrinogen beta chain (FGB), and fibrinogen gamma chain (FGG) between the two types of exosomes. As shown in Figure 3A, WJ-MSC exosomes showed higher abundances of FGA, FGB, and FGG than AD-MSC exosomes. To further illustrate the potential differences in exosome functions, the complement and coagulation cascades of WJ-MSC exosome-specific proteins were mapped. In addition to fibrinogen-related proteins, complement components C3 and C4, fumarate hydratase (FH), tissue-type plasminogen activator (tPA), alpha-1-antitrypsin (A1AT), and clusterin were more abundant in WJ-MSC exosomes than in AD-MSC exosomes (red boxes, Figure 3B). Since the complement and coagulation cascade pathway is involved in many stages of wound healing [25,26], fibrinogen was selected for further in vitro cell assays.

### 2.5. Effects of WJ-MSC Exosomes on Keratinocyte Growth

**Figure 4:**
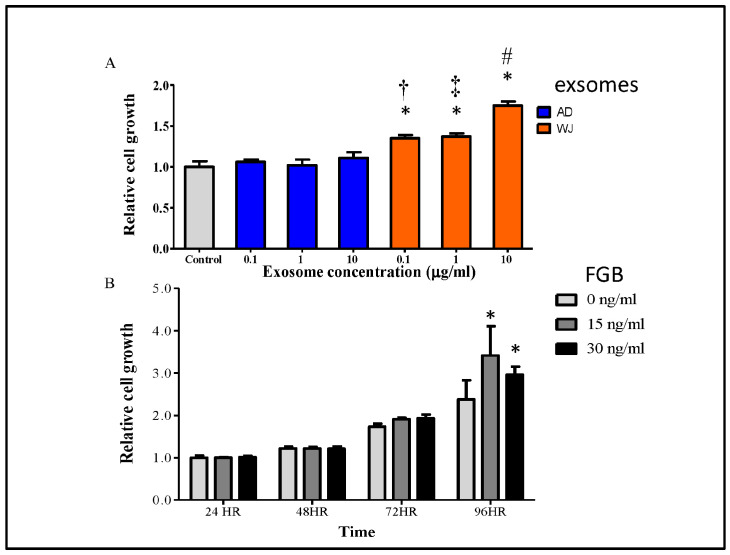
Effects of exosomes and FGB on keratinocyte proliferation. (**A**) HaCaT cells (0.5 × 10^3^ mL^−1^) were treated with/without AD-MSC or WJ-MSC exosomes. Cell proliferation was determined at 48 h. (The number of independent experiment repetitions was tightly controlled at least three times. For each repetition, triplicates were used.) (**B**) HaCaT cells were treated with/without different concentrations of recombinant FGB protein in a 96-well plate, and cell proliferation was determined at 24, 48, 72, and 96 h. * *p* < 0.05, compared with the control group. (The number of independent experiment repetitions was tightly controlled at least three times. For each repetition, triplicates were used.) ^†^ *p* < 0.05, compared with the AD 0.1 μg·mL^−1^ group. ^‡^ *p* < 0.05, compared with the AD 1 μg·mL^−1^ group. ^#^ *p* < 0.05, compared with the AD 10 μg·mL^−1^ group. Abbreviations: AD-MSCs, adipose tissue-derived mesenchymal stem cells; WJ-MSCs, Wharton’s jelly-derived mesenchymal stem cells; FGB, fibrinogen β chain.

WJ-MSC exosomes have been reported to promote skin wound healing [27]. Since WJ-MSC exosomes showed more abundant wound-healing-related proteins, we examined their effects on keratinocyte growth. Human HaCaT cells (0.5 × 10^3^·mL^−1^) were treated with or without exosomes at indicated concentrations and time points in a 96-well plate. In the WST-1 assay, HaCaT cells showed significantly enhanced proliferation when treated with WJ-MSC exosomes compared with those treated with AD-MSC exosomes at 48 h (Figure 4A). In addition, the cell proliferation-promoting effect was also assessed by using the recombinant proteins of the fibrinogen protein family. Among the three detected fibrinogen-related proteins, FGA and FGB reached significant differences, but FGG did not. In addition, the FGB recombinant protein was commercially available to us. Therefore, we used FGB for in vitro cell assays. As shown in Figure 4B, the recombinant FGB protein promoted HaCaT cell proliferation at 96 h. Thus, WJ-MSC exosomes better promoted keratinocyte growth than AD-MSC exosomes, consistent with the higher abundance of fibrinogen proteins in the former type.

### 2.6. Effects of WJ-MSC Exosomes on Keratinocyte Migration

**Figure 5:**
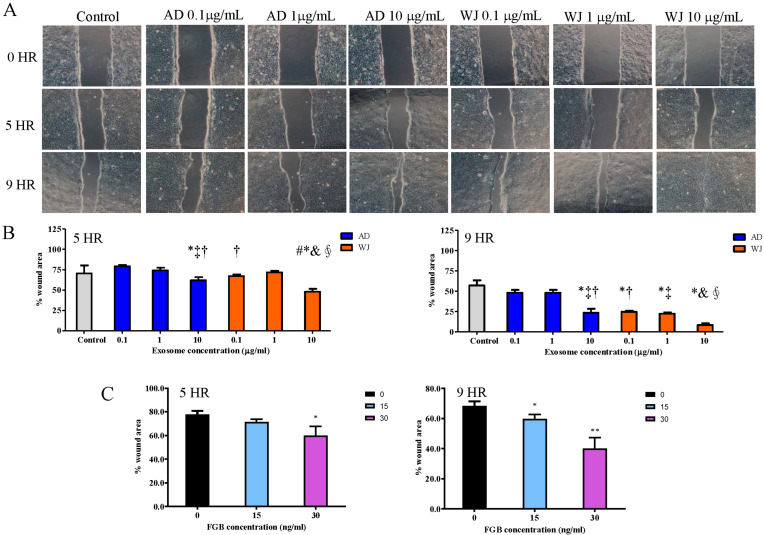
Time-dependent microscopic images of HaCaT cell-free gaps after treatment with/without AD-MSC and WJ-MSC exosomes and FGB recombinant protein. (**A**) Time-dependent microscopic images of HaCaT cell-free gaps after treatment with/without AD-MSC and WJ-MSC exosomes for 48 h (200×). (The number of independent experiment repetitions was tightly controlled at least three times). (**B**) The quantification results of wound-healing assay. The “% wound area” was normalized based on the control set at 0 h set. For each repetition, triplicates were used). * *p* < 0.05, compared with the control group. ^†^ *p* < 0.05, compared with the AD 0.1 μg·mL^−1^ group. ^‡^ *p* < 0.05, compared with the AD 1 μg·mL^−1^ group. ^#^ *p* < 0.05, compared with the AD 10 μg·mL^−1^ group. ^∮^ *p* < 0.05, compared with the WJ 0.1 μg·mL^−1^ group. ^&^ *p* < 0.05, compared with the WJ 1 μg·mL^−1^ group. (**C**) FGB protein treatment also promoted cell migration of keratinocyte cells. The “% wound area” was normalized based on the 0 ng/mL at 0 h set. * and ** denoted *p* < 0.05 and *p* < 0.01, respectively. Abbreviations: AD-MSCs, adipose tissue-derived mesenchymal stem cells; WJ-MSCs, Wharton’s jelly-derived mesenchymal stem cells.

Moreover, the migration-promoting effects of MSC exosomes on HaCaT cells were explored using an in vitro wound-healing assay. After treatment at different concentrations, both MSC exosomes significantly enhanced HaCaT cell migration. However, WJ-MSC exosomes promoted HaCaT cell migration better than AD-MSC exosomes (Figure 5A,B). In addition, migration ability was also examined with keratinocyte treated with FGB recombinant protein. It turned out that the migration ability of keratinocyte was elevated with FGB protein treatment (Figure 5C).

**Figure 6:**
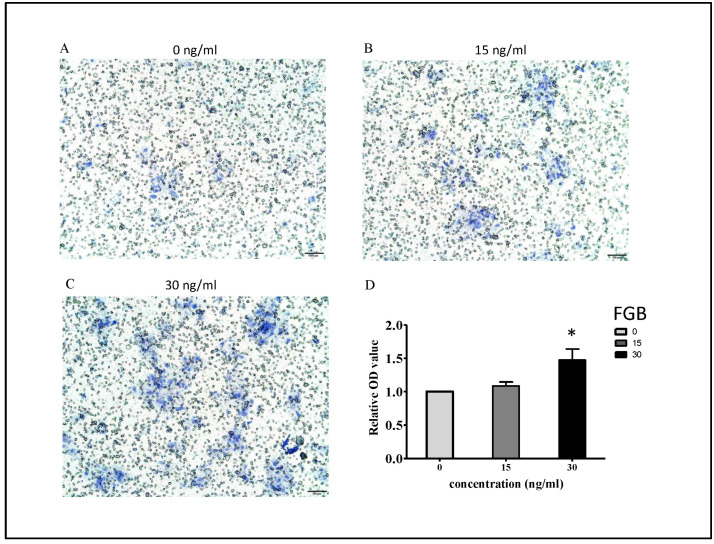
Trans-well migration assay on keratinocyte cells treated with FGB recombinant protein. Keratinocyte cells treated with different dosages of FGB protein were subjected to migration assays. (**A**–**C**) One run of migration assay demonstrated that more keratinocyte cells migrated to the down size of trans-well membrane with higher FGB administration (100×). (**D**) Through three independent assays, FGB treatment (30 ng/mL) significantly (*p*-value < 0.05) enhanced the migration ability of keratinocyte cells. * *p* < 0.05, compared with the control group, 0 ng/mL. Abbreviations: FGB, fibrinogen β chain.

In addition, we also examined the migration-promoting effects of FGB with the trans-well migration assay. As shown in Figure 6A–C, with higher FGB administration, more HaCaT cells penetrated the membrane, migrating to the down size of the membrane (one run of trans-well migration assay). Through three independent assays, HaCaT cells treated with 30 ng/mL of FGB showed significantly elevated migration ability (Figure 6).

## 3. Discussion

In the present study, we compared the exosome protein profiles of two common types of stem cells, AD-MSCs and WJ-MSCs. The functions of WJ-MSC exosome-specific proteins were mainly related to cell biological processes, while those of AD-MSC exosome-specific proteins were mainly related to neurodegenerative diseases. This finding implies that exosomes derived from these two different types of stem cells may serve distinct functions. WJ-MSC exosomes contained significantly more abundant fibrinogen and other key proteins related to the complement and coagulation pathways. Previous reports have indicated that WJ-MSC exosomes significantly inhibit apoptosis-inducing factor (AIF) nuclear translocation, poly (ADP-ribose) polymerase-1 (PARP1) activation, and H_2_O_2_-induced apoptosis in keratinocytes [28]. They can also promote keratinocyte migration; enhance skin wound healing, skin barrier formation, and angiogenesis; and reduce scar formation [28,29]. These results suggest that the direct administration of hucMSC-Ex could be a promising and effective treatment for skin wounds, offering significant clinical value. Our study results showed that WJ-MSC exosomes more effectively promote the proliferation and migration of keratinocytes than AD-MSC exosomes, possibly partially mediated through their higher FGB content. However, a limitation of our study is the lack of an animal model demonstrating the in vivo effectiveness of WJ-MSC-derived exosomes in facilitating wound healing. MSCs are pluripotent stem cells with self-renewal and differentiation ability. Umbilical cord-derived MSCs are the most original MSCs, and they are effective against several autoimmune diseases, such as lupus erythematosus, psoriasis, and rheumatoid arthritis [30,31,32]. AD-MSCs possess potent differentiation ability and exhibit potential for wound healing and treatment of organ injury [33,34,35]. Despite the extensive clinical significance of umbilical cord-derived MSCs and AD-MSCs, their applications are somewhat different. Although the functions of MSCs have been verified in regenerative medicine and autoimmune diseases, several limitations remain in their clinical application, such as source, stability, and ethical issues [36]. Compared with MSCs, exosomes are more stable and can be retained in the host after allogeneic implantation because of the weaker host-versus-graft reaction [37]. MSC-derived exosomes serve immune regulatory and regenerative functions similar to MSCs [38,39]. Therefore, exosomes derived from MSCs can offer alternative therapeutic options for various diseases. For their safety and efficacy, MSC-derived exosomes are currently being evaluated in clinical trials targeting various diseases, including immune-mediated diseases (e.g., dry eye in GVHD, type 1 diabetes mellitus, and periodontitis), wound healing (e.g., epidermolysis bullosa and idiopathic holes in retina), neurological diseases (e.g., Alzheimer’s disease), cardiovascular diseases (e.g., acute myocardial infarction, stroke, hypoxic pulmonary hypertension), and cancer (e.g., breast, gastric, and pancreatic cancers) [40,41].

Fibrinogen is a hepatogenic glycoprotein complex circulating in the blood [42]. When injury occurs, fibrinogen is converted by thrombin to fibrin, and a fibrin-based blood clot stops bleeding. In addition, fibrin mediates platelet and endothelial cell interaction, fibroblast proliferation, and angiogenesis, thereby promoting revascularization and wound healing [43]. Wound healing is a complex process, including hemostasis, inflammation, proliferation, and remodeling [26]. In addition, the fibrin matrix plays other key roles in tissue repair. For instance, fibrin is an important part of the matrix, which can protect the underlying tissue and support the migration and proliferation of inflammatory, endothelial, and stromal cells involved in tissue repair. An increasing cell growth rate was observed from 72 h in FGB-treated cells, and there was no significant difference at 48 h (Figure 4B). However, in cells treated with exosomes (Figure 4A), increased cell proliferation was observed during exosome treatment extracted from WJ-MSCs at 48 h. Our experimental results indicated that there may be other proteins or RNA molecules that can induce cell proliferation. However, in vivo experiments to support our point need to be carried out.

Wound healing potential decreases with aging and in the presence of certain condition, such as diabetes and malnutrition. In an in vitro model of wound healing, AD-MSC exosomes have been shown to prompt cell proliferation and migration and inhibit cell apoptosis via the Wnt/beta-catenin signaling pathway [44]. Similarly, another study showed that AD-MSC exosomes promoted fibroblast proliferation and migration and optimized collagen deposition via the PI3K/Akt signaling pathway to further accelerate wound healing [45]. WJ-MSCs are stem cells derived from the subepithelial layer of the umbilical cord. WJ-MSCs exhibit a multilineage potential and can differentiate into diverse cell types, facilitating osteo-, chondro-, adipo-, and cardio-genesis [46]. Furthermore, WJ-MSCs possess strong proliferative potential and potent immunoregulatory features, making them promising for application in regenerative medicine. In the present study, fibrinogen proteins were more abundant in WJ-MSC exosomes than in AD-MSC exosomes. Therefore, WJ-MSC exosomes may also hold immense potential for wound healing given their higher fibrinogen content. Moreover, MSC exosomes better promoted keratinocyte proliferation and migration than AD-MSC exosomes. Exosomes derived from cord blood MSCs and AD-MSCs have been compared in recent studies [6,47]. Here, we compared the protein components between WJ-MSC and AD-MSC exosomes, providing important information for stem cell therapy. The exploration of new biological products or materials as alternatives to mesenchymal stem cells to elucidate their regenerative repair functions and mechanisms has emerged as a significant focus in regenerative medicine research. Clarifying specific key proteins in exosomes could facilitate the development of mesenchymal stem cell exosome-based treatments for diseases relevant to clinical patients.

In this study, FGB recombinant protein was applied to treat keratinocyte cells at dosages of 0, 15, and 30 ng/mL. In another study, we examined the concentration of serum FGB protein in subjects. It turned out that serum FGB is approximately 15 ng/mL in healthy control subjects. Therefore, we treated keratinocyte cells with these dosages.

## 4. Materials and Methods

### 4.1. Isolation of AD-MSC and WJ-MSC Exosomes

Human AD-MSCs and WJ-MSCs were purchased from the Cellular Engineering Technologies (CET) Research Laboratories (Coralville, IA, USA). The cells were maintained in SF1 hMSC medium (Unimed Healthcare Inc., Taipei, Taiwan) in a humidified chamber at 37 °C and 5% CO_2_ until 80% confluent. For cell passage, the cells were washed with phosphate-buffered saline (PBS) and then treated with trypsin (TrypLE™ Select; Thermo Fisher Scientific, Waltham, MA, USA). The trypan blue exclusion method was used to evaluate cell viability. For flow cytometric identification, WJ-MSCs and/or AD-MSCs were stained with a human MSC marker antibody panel (BD Pharmingen system, Franklin Lakes, NJ, USA), including anti-human CD34-FITC, CD45-BV786, CD14-FITC, CD90-PE, CD44-PE, CD20-PE, CD105, CD166-PE, and CD73-PE, and subjected to flow cytometry (BD FACSCalibur™).

Exosomes were purified from the conditioned media of AD-MSCs and WJ-MSCs using a sequential filtration method. In brief, conditioned media were collected from AD-MSCs or WJ-MSCs (within passage 7) cultured in a serum-free medium at 37 °C in a humidified atmosphere of 5% CO_2_ for 24–48 h. Cells, debris, and other large particles were removed by centrifugation and filtration using a 0.22 μm filter. Exosomes were further concentrated with centrifugal filters (Vivaspin-20; MWCO, Sartorius, Tokyo, Japan) and then purified using the ExoQuickTC exosome precipitation solution according to the manufacturer’s recommendations. Protein concentration of the purified exosomes was determined using the bicinchoninic acid assay (BCA) protein assay (Thermo Fisher Scientific, Carlsbad, CA, USA). Next, the particle size and concentration of exosomes were determined using a ZetaView PMX 120 V4.1 instrument (Particle Metrix GmbH, Ammersee, Bavaria, Germany) according to the manufacturer’s protocol. For each sample, five 20 s videos were recorded at camera level = 11. Analysis was performed at detection limit = 3. The size and concentration analyses were performed blind, and samples from visits 1 and 2 were processed simultaneously. All samples were analyzed with the same settings using NTA 3.0 to enhance concentration measurement accuracy. Western blotting was performed to characterize the exosome surface markers: CD9 (Abcam, Cambridge, MA, USA), CD63 (Systems Biosciences, Palo Alto, CA, USA), and TSG101 (internal marker; Abcam).

### 4.2. Western Blotting

The isolated exosomes were subjected to protein enrichment. Briefly, protein samples prepared from the isolated exosomes were lysed in a RIPA buffer (Cell Signaling Technology, Danvers, MA, USA) and supplemented with a protease inhibitor cocktail (Roche, Mannheim, Germany). Subsequently, total proteins (40 μg) were resolved on a 12% polyacrylamide gel and transferred onto polyvinylidene fluoride membranes using a semidry system. The membranes were blocked with 5% skim milk (BD Biosciences, Franklin, NJ, USA) in TBST for 1 h at room temperature and then incubated with primary antibodies at 4 °C overnight. After washing and incubating with the horseradish peroxidase (HRP)-conjugated secondary antibodies, the blots were visualized using enhanced chemiluminescence (ECL) detection reagents (DoGenBio, Seoul, Republic of Korea) and exposed onto X-ray films.

### 4.3. Isobaric Tags for Relative and Absolute Quantitation (iTRAQ) Gel-Free Proteomics

iTRAQ gel-free proteomics were selected to globally screen and compare the protein contents of exosomes derived from AD-MSCs and WJ-MSCs, as reported previously [48]. In brief, the collected exosome protein samples were first subjected to high-abundance protein depletion with the Pierce Top 12 Abundant Protein Depletion Spin Columns (85165; Thermo Fisher Scientific). Then, the protein samples from AD-MSCs and WJ-MSCs were prepared using the iTRAQ Reagents Multiplex Kit (4352135; Sciex, Framingham, MA, USA). After a standard quality check, labeled peptide samples were determined using LC/Q-Exactive Orbitrap MS (Thermo Fisher Scientific) and analyzed using Proteome Discoverer v2.4 (Thermo Fisher Scientific) with reference to the MASCOT 2.5 database (Matrix science, Portland, OR, USA) to determine the relative abundances of the detected proteins.

### 4.4. Culture and Growth of Keratinocytes

Human keratinocyte HaCaT cells (Procell Life Science and Technology Co., Ltd., Wuhan, China) were cultured in a minimal essential medium (MEM; cat. no. PM150410, Procell) with 15% fetal bovine serum, 100 U·mL^−1^ penicillin, and 100 µg·mL^−1^ streptomycin at 37 °C with 95% air and 5% CO_2_.

### 4.5. Cell Proliferation Assay

The cultured cells (0.5 × 10^3^·mL^−1^) were seeded for 24 h in a culture medium. Then, the cells were treated with/without purified exosomes (0, 0.1, 1, and 10 µg·mL^−1^) for 24, 48, and 96 h in a 96-well plate. In other experiments, the cells were pretreated with/without the recombinant fibrinogen beta chain (FGB) (cat. no. pro-2210, ProSpec, Rehovot, Israel) at 0, 15, or 30 ng/mL. Cell proliferation was assessed using water-soluble tetrazolium salt (WST-1) assays (Roche Applied Science, Indianapolis, IN, USA) according to the manufacturer’s instructions.

### 4.6. Wound Healing Assay

To determine the wound-healing properties of exosomes, a cell migration assay was performed using HaCaT keratinocytes. The migration assay was performed using a culture-insert 2 well in l dish of 35 mm (Ibidi, 81176, Germany). In brief, the cells were seeded into a culture-insert 2 well (5 × 10^4^ cells·70 μL^−1^ per well) and incubated for 24 h in a growth medium with purified exosomes (0, 0.1, 1, and 10 µg·mL^−1^) until 95–100% cell density was reached. Then, the biocompatible silicone gasket was removed to create a cell-free gap in which the cell migration could be visualized. Thereafter, the culture dish was filled with 3 mL of medium. Time-dependent cell migration and wound healing in a cell-free gap were assessed under an inverted microscope at ×10 magnification (Leica DMI3000 B; Leica, Solms, Germany). Open areas were determined using Moticam 1080.

### 4.7. Trans-Well Migration Assay

We conducted a trans-well migration assay by referring to a previous study (PMID: 36361536). Human keratinocyte HaCaT cells were pretreated with a recombinant fibrinogen beta chain (FGB) (cat. no. pro-2210, ProSpec) at 0, 15, or 30 ng·mL^−1^ for 24 h. Then, 2 × 10^5^ cells were seeded onto the upper chamber (insert) of the 24-well trans-well plates (Millipore, MCSP24H48, Burlington, MA, USA), followed by incubation at 37 °C in 5% CO_2_ for 16 h. Then, the media and cells remaining at the top of the insert membrane were removed with a cotton-tipped swab. Next, the membranes were fixed with 100% methanol for 30 min, washed with PBS, and stained with crystal violet (Sigma, Kawasaki, Japan) for 30 min. Finally, the stained cells were collected and dissolved in 10% acetic acid and measured at a wavelength of 590 nm on an ELISA reader.

## 5. Conclusions

Exosomes derived from AD-MSCs and WJ-MSCs exhibit similar surface protein markers. The shared proteins suggest their common properties. However, significant differential proteins were identified between AD-MSC and WJ-MSC exosomes. The different proteins between AD-MSC and WJ-MSC exosomes imply their distinct potential functions. Unveiling the various components of AD-MSC and WJ-MSC exosomes may help determine their different clinical applications.

## Figures and Tables

**Table 1:** The top 10 most significant pathways enriched by WJ up-regulated, AD up-regulated, or consensus proteins. We had the 315 (AD specific), 362 (WJ specific), and 1018 (consensus) proteins subjected to pathway enrichment analyses, and the top 10 most significant pathways of individual sets were tabulated.

Category	Pathway	*p*-Value
WJ	Complement and coagulation cascades	2.45 × 10^−9^
WJ	ECM–receptor interaction	2.11 × 10^−7^
WJ	Focal adhesion	1.05 × 10^−6^
WJ	Amoebiasis	4.06 × 10^−6^
WJ	Proteasome	2.98 × 10^−5^
WJ	Glycosaminoglycan biosynthesis—chondroitin sulfate/dermatan sulfate	2.37 × 10^−4^
WJ	Hypertrophic cardiomyopathy (HCM)	3.55 × 10^−4^
WJ	Dilated cardiomyopathy (DCM)	5.92 × 10^−4^
WJ	Staphylococcus aureus infection	1.38 × 10^−3^
WJ	PI3K-Akt signaling pathway	1.39 × 10^−3^
AD	Parkinson’s disease	5.63 × 10^−9^
AD	Protein digestion and absorption	3.02 × 10^−8^
AD	Cardiac muscle contraction	3.87 × 10^−8^
AD	Alzheimer’s disease	3.95 × 10^−8^
AD	Oxidative phosphorylation	2.76 × 10^−6^
AD	Huntington’s disease	2.24 × 10^−5^
AD	Metabolic pathways	6.67 × 10^−5^
AD	ECM–receptor interaction	1.19 × 10^−4^
AD	Carbon metabolism	2.16 × 10^−4^
AD	Thermogenesis	2.57 × 10^−4^
Consensus	Ribosome	1.56 × 10^−30^
Consensus	Proteasome	5.58 × 10^−13^
Consensus	Bacterial invasion of epithelial cells	1.66 × 10^−9^
Consensus	Carbon metabolism	9.65 × 10^−9^
Consensus	Protein processing in endoplasmic reticulum	2.41 × 10^−8^
Consensus	Endocytosis	5.19 × 10^−8^
Consensus	Pathogenic *Escherichia coli* infection	5.63 × 10^−8^
Consensus	Phagosome	1.58 × 10^−7^
Consensus	Biosynthesis of amino acids	1.63 × 10^−7^
Consensus	Regulation of actin cytoskeleton	2.09 × 10^−6^

## Data Availability

All the data are presented in the Supplementary Materials.

## Supplementary Materials

The supporting information can be downloaded at: https://www.mdpi.com/article/10.3390/ijms25094758/s1.
